# Bio-Mechano-Therapy for Refractory Patellar Tendinopathy in Athletes: A Randomized Comparative Study of Platelet-Rich Plasma, Extracorporeal Shock Wave Therapy, and Their Combination

**DOI:** 10.7759/cureus.106398

**Published:** 2026-04-03

**Authors:** Yuki Shimizu

**Affiliations:** 1 Orthopaedic Sports Medicine, Nippon Sports Science University, Tokyo, JPN; 2 Orthopaedic Sports Medicine, The Yokohama Front Bayside Clinic, Yokohama, JPN; 3 Orthopaedic Sports Medicine, Inagi Hirao Orthopaedic Clinic, Tokyo, JPN

**Keywords:** bio-mechano-therapy, extracorporeal shock wave therapy (eswt), orthopedic sports medicine, platelet-rich plasma (prp) therapy, randomized comparative study, refractory patellar tendinopathy, regenerative medicine, synergistic effect, tendon healing, ultrasound and mri evaluation

## Abstract

Background

Refractory patellar tendinopathy in competitive athletes frequently results in persistent pain and prolonged functional limitation despite structured rehabilitation programs. Platelet-rich plasma (PRP) and extracorporeal shock wave therapy (ESWT) have each demonstrated clinical efficacy; however, the potential synergistic effect of their combination, conceptualized as bio-mechano-therapy, remains insufficiently investigated in randomized clinical settings.

Purpose

To compare the clinical and structural outcomes of PRP alone, ESWT alone, and combined PRP plus ESWT therapy in athletes with refractory patellar tendinopathy.

Study design

This was a randomized comparative study with Level 2 evidence.

Methods

Ten competitive athletes with chronic patellar tendinopathy were randomly assigned to PRP alone (n = 5), ESWT alone (n = 2), or combined PRP+ESWT therapy (n = 3). All participants underwent a standardized eccentric rehabilitation program.

Clinical outcomes included visual analog scale (VAS), Victorian Institute of Sport Assessment-Patella (VISA-P), International Knee Documentation Committee (IKDC), Lysholm score, and Knee injury and Osteoarthritis Outcome Score (KOOS Total). Structural changes were assessed using magnetic resonance imaging (MRI) and ultrasonography at baseline and one, three, and six months.

Longitudinal clinical changes were analyzed using linear mixed-effects models including fixed effects for time, treatment group, and time-by-group interaction, with participants treated as random effects. Imaging improvement rates were compared using the Kruskal-Wallis test with Holm-adjusted post hoc analysis.

Results

Significant time effects were observed for all clinical outcomes (P < 0.01), whereas no significant time-by-group interactions were identified. Functional improvements were observed across all treatment groups, with no statistically significant between-group differences in patient-reported clinical scores.

Quantitative imaging analyses demonstrated significant between-group differences in ultrasonographic hypoechoic lesion area and intratendinous lesion length at six months (P = 0.031 and P = 0.015, respectively), favoring the PRP+ESWT group. MRI-based assessments showed progressive structural improvement over time, with a significant between-group difference identified for axial lesion thickness at six months (P = 0.046), whereas sagittal lesion length and axial lesion width did not differ significantly among groups. Effect sizes for ultrasonographic outcomes were moderate to large (ε² = 0.34-0.37).

All athletes returned to sport within six months, with no significant differences in return-to-play status among treatment groups.

Conclusion

Combined PRP and ESWT therapy demonstrated greater structural improvement on ultrasonographic assessment and numerically enhanced symptomatic recovery compared with monotherapy, although functional intergroup differences were not statistically significant. These findings suggest a potential synergistic interaction between biological stimulation and mechanotransductive loading in tendon healing.

Clinical relevance

Bio-mechano-therapy may represent a promising conservative treatment strategy for refractory patellar tendinopathy by promoting structural remodeling alongside clinical recovery in competitive athletes.

## Introduction

Refractory patellar tendinopathy is a common overuse condition among athletes who repeatedly perform jumping, acceleration, and deceleration [1]. Patellar tendinopathy is characterized by tendon degeneration, collagen disorganization, and neovascularization resulting from repetitive mechanical loading. These pathological changes contribute to persistent pain and functional impairment in athletes. When symptoms become chronic, the disorder can lead to substantial deterioration in athletic performance and prolonged time loss [2,3]. In elite athletes, persistent pain is not merely a symptomatic issue; it is a clinically important problem that directly restricts training volume, destabilizes performance, and may ultimately shorten athletic careers [4,5]. Accordingly, establishing an effective conservative strategy that achieves not only pain control but also high-quality recovery accompanied by structural restoration of tendon tissue remains a major priority in sports medicine.

Conservative management is widely regarded as first-line treatment for patellar tendinopathy, and eccentric exercise is considered the gold standard because mechanical loading can induce collagen realignment and tendon remodeling through mechanobiological stimulation of tenocytes [6,7]. However, in cases with chronic degeneration (tendinosis), histopathologic changes such as angiofibroblastic proliferation and disruption of the extracellular matrix may progress, and the biological healing response can become stalled. In these “treatment-resistant” cases, conventional exercise-based rehabilitation and physical modalities alone may be insufficient to adequately restart the repair cascade, resulting in persistent symptoms [3,8,9].

To address this challenge, platelet-rich plasma (PRP) has received increasing attention as a biologic intervention. PRP contains high concentrations of growth factors, including platelet-derived growth factor BB (PDGF-BB), transforming growth factor-β1 (TGF-β1), and vascular endothelial growth factor (VEGF), and has been shown to promote tenocyte proliferation, collagen synthesis, and extracellular matrix formation [10]. Clinically, PRP has been reported to improve pain and function in patellar tendinopathy [11]. Nevertheless, delayed onset of effect and substantial inter-individual variability in treatment response remain important limitations.

Extracorporeal shock wave therapy (ESWT) can induce angiogenesis-related mediators (eg, VEGF and endothelial nitric oxide synthase (eNOS)) through mechanotransduction [12] and has been shown to improve pain and Victorian Institute of Sport Assessment-Patella (VISA-P) scores in chronic tendon disorders [13]. ESWT has also been reported to exert analgesic effects by modulating nociceptive nerve terminals and improving local perfusion. However, ESWT monotherapy may have limitations in the extent of structural repair achieved and in long-term functional outcomes.

Recently, combined PRP and ESWT has been suggested to yield earlier pain reduction than either modality alone [14]. Moreover, ESWT may suppress the post-PRP upregulation of inflammatory alarmins (S100A8/A9), thereby normalizing the local microenvironment [15]. However, these data remain fragmented, and there is a lack of an integrated theoretical framework that captures interactions between biological and mechanical stimuli. Prospective studies that simultaneously evaluate clinical outcomes and imaging-based structural indices are also scarce.

Importantly, the combination of PRP and ESWT should not be viewed as merely additive. ESWT may enhance intracellular delivery of growth factors via shock wave-induced sonoporation and may physically amplify the release of platelet-derived mediators, thereby generating a “biological-mechanical synergistic effect (bio-mechano-synergy)” [16-19]. In this context, ESWT may “prime” the repair environment and increase tissue responsiveness to PRP-derived biological signals, potentially reactivating a stalled tendon repair cascade. We define this integrated concept as “bio-mechano-therapy.”

The purpose of this randomized controlled study was to compare PRP monotherapy, ESWT monotherapy, and combined therapy in athletes with refractory patellar tendinopathy, and to comprehensively evaluate the effects of bio-mechano-therapy on pain and functional outcomes as well as structural indices assessed by MRI and ultrasonography. Our primary objective was to clarify how interactions between biological and mechanical stimuli contribute to clinical recovery and tendon tissue repair.

## Materials and methods

Study design

This study was conducted as a prospective randomized comparative study to compare the therapeutic effects of ESWT, PRP, and combined PRP plus ESWT therapy for athletes with chronic patellar tendinopathy. Participants who met the inclusion criteria were randomly assigned to one of three treatment groups: PRP therapy, ESWT therapy, or combined PRP plus ESWT therapy. Randomization was performed using a computer-generated allocation sequence.

The primary outcome of the study was improvement in the VISA-P score [20] at three months. Secondary outcomes included pain intensity assessed using the visual analog scale (VAS) and functional outcomes evaluated using validated patient-reported outcome measures, including the Knee Injury and Osteoarthritis Outcome Score (KOOS) [21], the International Knee Documentation Committee (IKDC) subjective knee form [22], and the Lysholm Knee Scoring Scale [23].

The study was performed at the Nippon Sport Science University Clinic between June 2, 2021, and March 1, 2026.

The study protocol was approved by the Ethics Review Committee of Nippon Sport Science University (Approval No. 021-H028). Written informed consent was obtained from all participants prior to enrollment. The study was conducted in accordance with the Declaration of Helsinki.

Participants

Competitive athletes aged 18-50 years who presented to our sports orthopaedic outpatient clinic and were diagnosed with chronic patellar tendinopathy by a sports orthopaedic specialist were eligible for inclusion.

The diagnosis of patellar tendinopathy was established based on clinical symptoms, physical examination findings, and imaging confirmation. Clinical findings included localized tenderness at the inferior pole of the patella and pain during loading activities such as jumping, squatting, or resisted knee extension. Imaging confirmation was obtained using ultrasonography and/or magnetic resonance imaging (MRI) demonstrating characteristic tendon abnormalities.

Eligibility criteria

Participants were required to meet both of the following criteria: 1. Localized tenderness at the inferior pole of the patella or activity-related pain during knee extension or jumping tasks; and 2. At least one morphologic abnormality consistent with chronic patellar tendinopathy on ultrasonography or MRI, including hypoechoic tendon degeneration, intratendinous signal alteration, or tendon thickening.

Exclusion criteria

Exclusion criteria included contraindications to PRP or ESWT (eg, diabetes mellitus, rheumatoid arthritis, active infection, coagulopathy, immunosuppression, hemoglobin <11 g/dL, platelet count <150,000/mm³, current anticoagulant therapy, pregnancy, or malignancy), knee surgery or intra-articular corticosteroid injection within the previous three months, use of nonsteroidal anti-inflammatory drugs within five days, allergy to local anesthetics, and concomitant knee disorders.

Sample size consideration

This study was designed as an exploratory pilot trial; therefore, a formal a priori power calculation was not performed before study initiation. The primary aim was feasibility assessment and estimation of treatment effects to inform future confirmatory studies.

To provide statistical justification, a post hoc sample size estimation was conducted using improvement in the VISA-P score at three months as the primary outcome.

Based on preliminary study data, the mean VISA-P improvement was 22.3 points with a standard deviation of 18.8. Using a significance level (α) of 0.05, a statistical power of 80%, and a two-tailed paired comparison, the estimated required sample size was approximately nine participants. Considering a potential dropout rate of approximately 10%, a target enrollment of 10 participants was determined to be appropriate for this exploratory pilot investigation.

Randomization

After written informed consent was obtained, participants were allocated by simple randomization to the ESWT group (n = 2), PRP group (n = 5), or PRP plus ESWT group (n = 3). To confirm post-randomization baseline balance, age and body mass index were compared using one-way analysis of variance (ANOVA), and sex distribution was compared using the Fisher-Freeman-Halton exact test.

Interventions

PRP Therapy

PRP was prepared using an ACP double-syringe system (Arthrex, Naples, FL, USA) according to the manufacturer’s protocol. Approximately 15 mL of venous blood was collected and centrifuged for five minutes to obtain leukocyte-poor PRP (LP-PRP). The PRP used in this study was classified as LP-PRP based on the absence of leukocyte concentration. The mean injected PRP volume was 5.18 ± 0.8 mL. Under ultrasound guidance, PRP was injected into the region of maximal tendon pathology using a 22-gauge needle. All injections were performed by an experienced sports orthopaedic surgeon. Injections were administered three times at two-week intervals. 

ESWT

ESWT was delivered using a diffuse-type shock wave device (Physio Shock Master; Sakai Medical Co., Ltd., Tokyo, Japan). A total of 2500 shocks were applied to the point of maximal tenderness at a frequency of 8 Hz and a pressure of 2.4-4.0 bar. Treatment was performed once weekly for three sessions.

Combined Therapy (PRP＋ESWT）

In the combined group, PRP and ESWT were administered within the same treatment period using identical numbers of sessions, intervals, and output settings as in the respective monotherapy protocols.

Standardized rehabilitation

To control for potential confounding effects of rehabilitation, all participants followed a standardized structured rehabilitation program. The program began two weeks after the final treatment session. The rehabilitation protocol included progressive loading exercises, eccentric strengthening of the quadriceps-patellar tendon complex, and neuromuscular training aimed at improving lower extremity kinetic chain function. Eccentric exercises were performed as three sets of 15 repetitions twice daily, five days per week. Loading initially began at approximately 10% of body weight and was progressively increased according to symptom tolerance. High-intensity sports activities were restricted during the treatment period. A stepwise return-to-sport progression was permitted after clinical evaluation and physician approval [6].

Clinical evaluation

Assessments were performed at baseline and at four, eight, 12, and 24 weeks (corresponding to one, two, three, and six months). Pain intensity was evaluated using a 100-mm VAS.

Functional outcomes were assessed using validated patient-reported outcome measures including the VISA-P [20], KOOS [21], IKDC [22], and Lysholm score [23]. The primary outcome measure was the change in VISA-P score from baseline. Secondary outcome measures included VAS pain score, KOOS, IKDC subjective score, and Lysholm score.

The VISA-P questionnaire consists of eight items assessing pain, function, and sports participation, yielding a total score ranging from 0 to 100, with higher scores indicating superior function. A change of approximately 13 points is considered clinically meaningful.

The KOOS evaluates five domains (Pain, Symptoms, Activities of Daily Living, Sport/Recreation, and Quality of Life), each normalized to a 0-100 scale, with higher scores representing better knee status. KOOS Total was calculated as the mean of all subscales.

The IKDC subjective score evaluates knee symptoms, daily function, and sports activity levels and is expressed on a 0-100 scale, with higher scores indicating better knee function.

The Lysholm score assesses knee stability and functional performance on a 0-100 scale; improvements of approximately 10 points are generally regarded as clinically significant.

For all clinical outcomes, absolute change from baseline (Δ value) and percentage improvement were calculated.

Imaging evaluation

Magnetic resonance imaging (MRI) was performed using a 1.5-T system (Echelon Oval; Hitachi, Japan). Short tau inversion recovery (STIR) sequences were acquired in both sagittal and axial planes to evaluate intratendinous pathology. In accordance with the quantitative assessment method described by Golman et al. [24], intratendinous hyperintense signal on STIR images was defined as a pathologic lesion. Maximum lesion length was measured on sagittal images, and maximum lesion width and thickness were measured on axial images using electronic calipers on a PACS workstation. All MRI examinations were performed using identical acquisition parameters at each time point. Image measurements were conducted by a single investigator using a standardized measurement protocol.

Ultrasonography was performed using a Sonimage MX1 system (Konica Minolta, Tokyo, Japan) equipped with a 3-11 MHz linear probe. Based on the quantitative assessment concept described by Golman et al., hypoechoic regions on B-mode images were defined as lesions [20]. The hypoechoic lesion area (mm²) and the intratendinous lesion length (mm) on the longitudinal view were measured using ImageJ software.

All images were obtained using the same device under identical imaging conditions. All measurements were performed by a single assessor who was blinded to group allocation.

Return-to-sport evaluation

At six months, a self-administered questionnaire was used to assess pain during daily activities, return to sport, return to preinjury level, pain during sport activity, and recurrence. Return to sport was defined as resumption of official training or competition.

Statistical analysis

Baseline continuous variables are presented as median (range) or mean ± standard deviation, as appropriate. Between-group comparisons of baseline continuous variables were performed using the Kruskal-Wallis test or one-way ANOVA when applicable. Categorical variables were analyzed using Fisher’s exact test or the Fisher-Freeman-Halton exact test.

Longitudinal changes in clinical outcome measures (VAS, IKDC, Lysholm, VISA-P, and KOOS Total scores) were analyzed using linear mixed-effects models, including fixed effects for time, treatment group, and time-by-group interaction, with participants treated as random effects. When significant between-group effects were detected, post hoc multiple comparisons were performed using Tukey’s honestly significant difference (HSD) test.

Within-group changes from baseline were evaluated using paired t tests.

For imaging outcomes, between-group comparisons of improvement rates were analyzed using ANOVA. When the overall ANOVA results were statistically significant, Tukey post hoc multiple comparison tests were applied. In addition, percentage improvement rates across groups at individual time points were compared using the Kruskal-Wallis test, followed by Holm-adjusted post hoc comparisons when appropriate.

Effect sizes were calculated using Cohen’s d for clinical outcomes and epsilon squared (ε²) for imaging outcomes.

All statistical analyses were performed using IBM SPSS Statistics version 29 (IBM Corp., Armonk, NY, USA). All tests were two-sided, and a P value < 0.05 was considered statistically significant.

## Results

Participant characteristics

All 10 participants completed the six-month follow-up, and no adverse events were observed. A total of 10 athletes (eight men and two women) were included in the analysis. The overall mean age was 19.5 ± 2.0 years, and the mean body mass index was 23.2 ± 3.7 kg/m². Baseline demographic characteristics are summarized in Table 1.

**Table 1:** Baseline characteristics of the study population. Baseline demographic characteristics of the overall study population and each treatment group, including extracorporeal shock wave therapy (ESWT), platelet-rich plasma (PRP), and combined PRP plus ESWT, are presented. Overall cohort data are expressed as mean ± standard deviation, whereas group-specific continuous variables are presented as median (range) due to the small sample size. Categorical variables are presented as counts. Between-group comparisons were performed to assess baseline comparability. Values are presented as mean ± standard deviation or median (range), as appropriate. † Intergroup comparisons for continuous variables were performed using the Kruskal–Wallis test. ‡ Sex distribution was analyzed using the Fisher–Freeman–Halton exact test for a 2 × 3 contingency table. Abbreviations: ESWT, extracorporeal shock wave therapy; PRP, platelet-rich plasma; BMI, body mass index. All statistical tests were two-sided, and a P value < 0.05 was considered statistically significant. Statistical analyses were performed using IBM SPSS Statistics version 29 (IBM Corp., Armonk, NY, USA).

Variable	Overall (n=10)	ESWT (n=2)	PRP (n=5)	PRP + ESWT (n=3)	P value
Age, years	19.5 ± 2.0	18.0 (18.0–18.0)	20.0 (17.0–23.0)	20.0 (20.0–21.0)	0.468†
BMI, kg/m²	23.2 ± 3.7	22.87 (22.34–23.39)	23.66 (20.08–32.95)	20.48 (20.48–22.31)	0.219†
Sex, male/female	8 / 2	2 / 0	3 / 2	3 / 0	0.667‡

Participants were competitive athletes involved in sports requiring repetitive jumping and loading activities. The sports represented in the cohort included soccer, basketball, volleyball, and athletics. Baseline characteristics were comparable across treatment groups, with no significant between-group differences in age (P = 0.468), body mass index (P = 0.219), or sex distribution (P = 0.667).

Clinical outcomes

**Figure 1:**
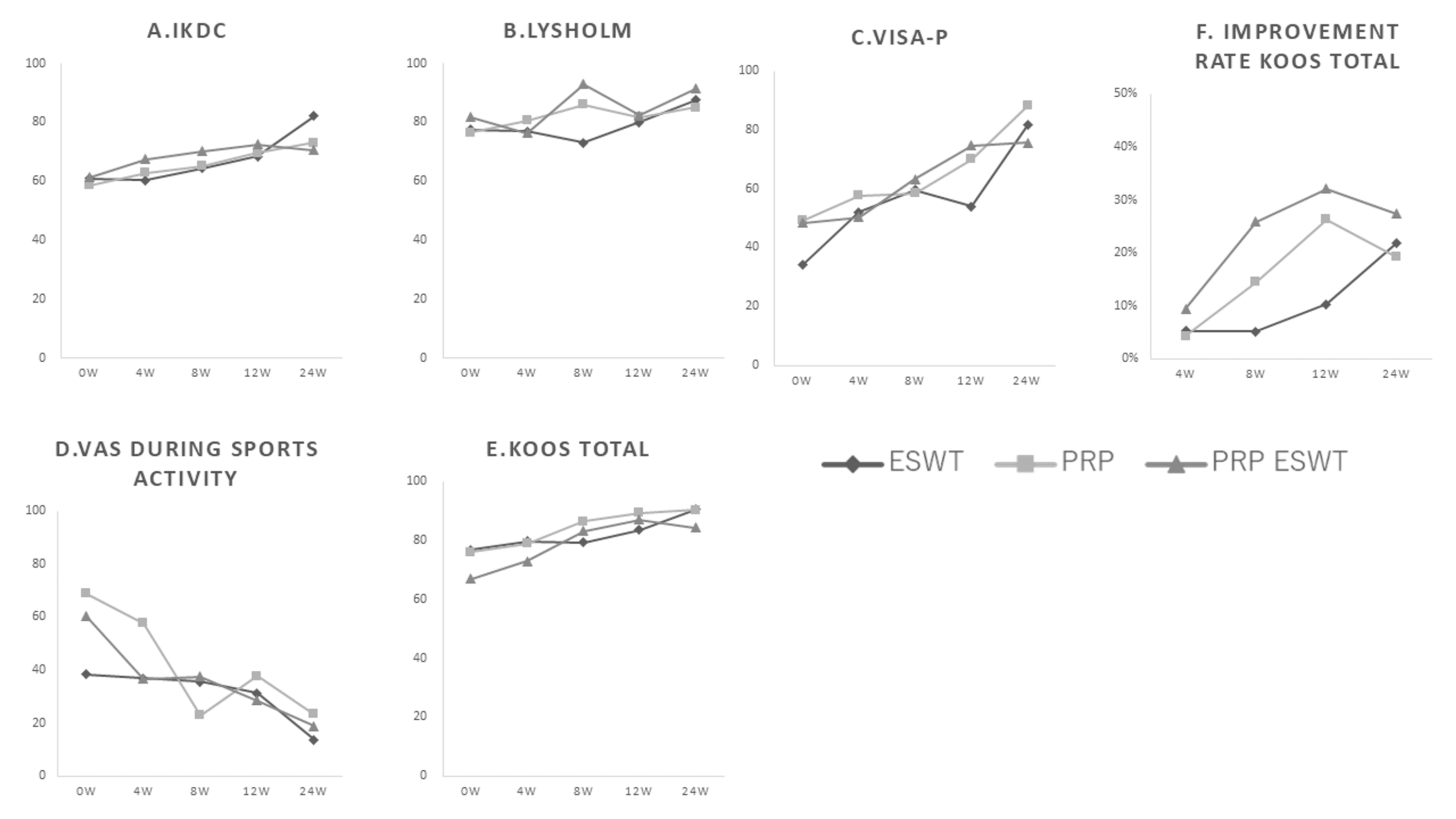
Longitudinal changes in clinical outcome scores and KOOS improvement rate among the three treatment groups. Temporal changes in (A) International Knee Documentation Committee (IKDC) score, (B) Lysholm score, (C) Victorian Institute of Sport Assessment–Patella (VISA-P) score, (D) visual analog scale (VAS) during sports activity, (E) Knee Injury and Osteoarthritis Outcome Score (KOOS Total), and (F) percentage improvement in KOOS Total are shown for the extracorporeal shock wave therapy (ESWT, n = 2), platelet-rich plasma (PRP, n = 5), and combined PRP plus ESWT (n = 3) groups. Assessments were performed at baseline and at four, eight, 12, and 24 weeks. Functional outcome scores progressively increased over time, whereas VAS scores decreased across all treatment groups, indicating clinical improvement. This figure illustrates longitudinal trends to facilitate visual comparison among treatment groups; detailed numerical data and statistical analyses are provided in Tables 2, 3. Data points represent group mean values at each time point. KOOS Total improvement rate was calculated as the percentage change from baseline. Abbreviations: ESWT, extracorporeal shock wave therapy; PRP, platelet-rich plasma; IKDC, International Knee Documentation Committee; VISA-P, Victorian Institute of Sport Assessment–Patella; KOOS, Knee Injury and Osteoarthritis Outcome Score; VAS, visual analog scale.

Pain and functional outcomes improved progressively over time across all treatment groups (Tables 2, 3, Figure 1).

**Table 2:** Longitudinal patient-reported outcome measures over six months. Patient-reported outcome measures, including visual analog scale (VAS), International Knee Documentation Committee (IKDC) score, Lysholm score, Victorian Institute of Sport Assessment–Patella (VISA-P), and Knee Injury and Osteoarthritis Outcome Score (KOOS Total), are presented for the extracorporeal shock wave therapy (ESWT, n = 2), platelet-rich plasma (PRP, n = 5), and combined PRP plus ESWT (n = 3) groups at baseline, one month, three months, and six months. Values are presented as mean ± standard deviation. Linear mixed-effects models included fixed effects for time, treatment group, and their interaction, with patient included as a random effect. P values for time effects indicate overall longitudinal changes across time points. Group main effect P values represent overall differences among treatment groups independent of time, whereas interaction P values indicate differences in longitudinal change patterns between groups. Abbreviations: ESWT, extracorporeal shock wave therapy; PRP, platelet-rich plasma; VAS, visual analog scale; IKDC, International Knee Documentation Committee; VISA-P, Victorian Institute of Sport Assessment–Patella; KOOS, Knee injury and Osteoarthritis Outcome Score.

Outcome	Group	Baseline	1 Month	3 Months	6 Months	Time Effect p	Group Main Effect p	Interaction p
VAS	ESWT (n=2)	38.5 ± 41.0	36.9 ± 22.4	31.3 ± 29.6	13.8 ± 6.0	<0.001	0.61	0.42
	PRP (n=5)	68.2 ± 20.8	57.8 ± 28.5	37.8 ± 19.0	23.0 ± 22.6			
	PRP + ESWT (n=3)	60.4 ± 18.5	36.6 ± 12.0	28.5 ± 20.4	18.9 ± 8.6			
IKDC	ESWT (n=2)	61.0 ± 1.6	60.4 ± 2.5	68.4 ± 7.3	82.2 ± 5.7	<0.001	0.74	0.31
	PRP (n=5)	58.6 ± 7.8	62.8 ± 6.1	70.6 ± 12.6	73.1 ± 8.4			
	PRP + ESWT (n=3)	61.3 ± 9.9	67.4 ± 5.1	72.4 ± 11.0	70.5 ± 11.7			
Lysholm	ESWT (n=2)	77.5 ± 2.1	77.0 ± 4.2	80.0 ± 5.7	87.5 ± 3.5	0.002	0.68	0.38
	PRP (n=5)	76.6 ± 6.7	80.6 ± 8.5	81.8 ± 19.7	85.0 ± 16.4			
	PRP + ESWT (n=3)	81.7 ± 5.0	76.3 ± 7.3	82.3 ± 9.7	91.3 ± 7.0			
VISA-P	ESWT (n=2)	34.0 ± 33.9	52.0 ± 17.0	54.0 ± 7.1	81.5 ± 0.7	<0.001	0.73	0.21
	PRP (n=5)	49.2 ± 9.4	57.2 ± 2.9	70.0 ± 20.2	88.2 ± 13.1			
	PRP + ESWT (n=3)	48.3 ± 19.4	50.3 ± 22.7	74.7 ± 16.6	75.7 ± 11.3			
KOOS Total	ESWT (n=2)	76.8 ± 20.2	79.7 ± 21.5	79.3 ± 24.7	90.5 ± 1.7	<0.001	0.79	0.18
	PRP (n=5)	78.1 ± 9.9	81.0 ± 9.7	88.5 ± 13.2	92.3 ± 5.8			
	PRP + ESWT (n=3)	73.6 ± 8.2	79.5 ± 8.5	89.8 ± 12.1	90.9 ± 7.6			

**Table 3:** Changes from baseline and effect sizes for patient-reported outcomes. Mean changes (Δ) from baseline at one, three, and six months are presented for visual analog scale (VAS), International Knee Documentation Committee (IKDC), Lysholm score, Victorian Institute of Sport Assessment–Patella (VISA-P), and Knee injury and Osteoarthritis Outcome Score (KOOS Total) across extracorporeal shock wave therapy (ESWT), platelet-rich plasma (PRP), and combined PRP plus ESWT groups. Effect sizes at six months were calculated using Cohen’s d to quantify the magnitude of treatment effects. Footnote: Values are expressed as mean ± standard deviation. Δ indicates change from baseline. Cohen’s d values represent within-group effect sizes at six months and were interpreted as small (0.2), medium (0.5), and large (≥0.8). Negative values indicate improvement for VAS (pain reduction), whereas positive values indicate improvement for IKDC, Lysholm, VISA-P, and KOOS Total. * The asterisk indicates an unusually large effect size resulting from extremely small within-group variability (very low standard deviation of change scores) in the ESWT group and should be interpreted with caution due to the small sample size (n = 2). Abbreviations: ESWT, extracorporeal shock wave therapy; PRP, platelet-rich plasma; VAS, visual analog scale; IKDC, International Knee Documentation Committee; VISA-P, Victorian Institute of Sport Assessment–Patella; KOOS, Knee injury and Osteoarthritis Outcome Score.

Outcome	Group	Δ1 Month	Δ3 Months	Δ6 Months	Cohen’s d (6M)
VAS	ESWT (n=2)	-1.6 ± 18.6	-7.2 ± 20.3	-24.7 ± 35.0	0.71
	PRP (n=5)	-10.4 ± 17.9	-30.4 ± 22.5	-45.2 ± 19.7	2.29
	PRP + ESWT (n=3)	-23.8 ± 10.2	-31.9 ± 16.1	-41.5 ± 14.8	2.80
IKDC	ESWT (n=2)	-0.6 ± 3.1	+7.4 ± 6.4	+21.2 ± 6.2	3.42
	PRP (n=5)	+4.2 ± 4.5	+12.0 ± 11.7	+14.5 ± 9.1	1.59
	PRP + ESWT (n=3)	+6.1 ± 6.2	+11.1 ± 10.8	+9.2 ± 12.4	0.74
Lysholm	ESWT (n=2)	-0.5 ± 3.0	+2.5 ± 4.8	+10.0 ± 4.2	2.38
	PRP (n=5)	+4.0 ± 7.2	+5.2 ± 18.3	+8.4 ± 15.0	0.56
	PRP + ESWT (n=3)	-5.4 ± 7.0	+0.6 ± 8.9	+9.6 ± 8.3	1.16
VISA-P	ESWT (n=2)	+18.0 ± 20.1	+20.0 ± 10.5	+47.5 ± 1.1	43.18*
	PRP (n=5)	+8.0 ± 4.8	+20.8 ± 17.3	+39.0 ± 14.0	2.79
	PRP + ESWT (n=3)	+2.0 ± 23.0	+26.3 ± 17.1	+27.4 ± 13.5	2.03
KOOS Total	ESWT (n=2)	+2.95 ± 7.57	+2.50 ± 10.32	+13.70 ± 19.37	0.71
	PRP (n=5)	+2.88 ± 7.56	+10.41 ± 11.73	+14.24 ± 6.49	2.19
	PRP + ESWT (n=3)	+5.93 ± 5.73	+16.23 ± 10.30	+17.37 ± 10.78	1.61

Linear mixed-effects models were constructed, including fixed effects for time, treatment group, and the time-by-group interaction, with participants treated as random effects. The analyses demonstrated significant time effects for all clinical outcome measures, including VAS, IKDC, Lysholm, VISA-P, and KOOS Total scores (all P ≤ 0.002), indicating overall longitudinal improvement irrespective of treatment modality.

In contrast, no significant group main effects were observed (VAS, P = 0.61; IKDC, P = 0.74; Lysholm, P = 0.68; VISA-P, P = 0.73; KOOS Total, P = 0.79), nor were significant time-by-group interaction effects detected (VAS, P = 0.42; IKDC, P = 0.31; Lysholm, P = 0.38; VISA-P, P = 0.21; KOOS Total, P = 0.18). These findings indicate that although clinical outcomes improved significantly over time, improvement patterns were comparable among treatment groups.

Pain outcome (VAS)

VAS scores during sports activity decreased across all groups throughout follow-up (Table 2, Figure 1D).

Baseline variability was relatively large in the ESWT group (38.5 ± 41.0), likely reflecting inter-individual variability associated with the small sample size (n = 2). Source data were rechecked and confirmed to be accurate.

At six months, mean changes from baseline were −24.7 ± 35.0 in the ESWT group, −45.2 ± 19.7 in the PRP group, and −41.5 ± 14.8 in the PRP plus ESWT group (Table 3). Effect sizes were moderate in the ESWT group (Cohen’s d = 0.71) and large in the PRP (d = 2.29) and combined groups (d = 2.80), although between-group differences were not statistically significant.

Functional outcomes

Functional outcome scores improved steadily across the observation period in all treatment groups (Table 2; Figure 1A-1C, 1E).

IKDC and Lysholm scores demonstrated progressive improvement from baseline to six months, accompanied by moderate-to-large effect sizes (Table 3). VISA-P scores showed marked functional recovery, with six-month mean improvements of +47.5 ± 1.1 in the ESWT group, +39.0 ± 14.0 in the PRP group, and +27.4 ± 13.5 in the PRP plus ESWT group.

KOOS Total scores also increased continuously over time. Improvements from baseline at three months were +2.50 ± 10.32 (ESWT), +10.41 ± 11.73 (PRP), and +16.23 ± 10.30 (PRP+ESWT), with further gains observed at six months (+13.70 ± 19.37, +14.24 ± 6.49, and +17.37 ± 10.78, respectively). Effect size analysis indicated moderate-to-large clinical effects across all treatment groups.

Overall findings

Overall, significant longitudinal improvements were observed across all treatment groups; however, neither group main effects nor interaction effects reached statistical significance despite numerically greater improvements in PRP-containing treatments.

MRI outcomes

Quantitative MRI analysis demonstrated time-dependent structural improvement across all treatment groups. At six months, significant between-group differences were identified for axial lesion thickness, whereas other MRI-derived parameters did not show statistically significant intergroup differences (Table 4).

**Table 4:** Between-Group Analysis of Imaging Improvement Rates (One-Way ANOVA) Improvement rate = percentage reduction from baseline Between-group comparisons of structural imaging improvement rates were analyzed using one-way analysis of variance (ANOVA) across treatment groups: extracorporeal shock wave therapy (ESWT; n = 2), platelet-rich plasma (PRP; n = 5), and combined PRP plus ESWT (n = 3). Improvement rates represent percentage reduction from baseline lesion measurements obtained using ultrasonography (US) and magnetic resonance imaging (MRI). F-statistics (F) with corresponding degrees of freedom [F(2,7)] and associated p-values are reported for each outcome. Sagittal MRI indicates measurements obtained on sagittal imaging planes, whereas axial MRI refers to measurements obtained on axial imaging planes. Values are expressed as mean ± standard deviation.

Outcome	Imaging Plane	Time Point	ESWT (n=2) Mean ± SD	PRP (n=5) Mean ± SD	PRP+ESWT (n=3) Mean ± SD	F-statistic	ANOVA p-value
US Lesion Area (mm²)	Axial	6 Months	0.315 ± 0.40	0.588 ± 0.11	0.874 ± 0.10	F(2,7)=5.90	0.031
US Lesion Length (mm)	Sagittal	6 Months	0.351 ± 0.19	0.390 ± 0.15	0.777 ± 0.10	F(2,7)=8.03	0.015
MRI Lesion Length (mm)	Sagittal	6 Months	0.488 ± 0.15	0.387 ± 0.23	0.411 ± 0.04	F(2,7)=0.22	0.811
MRI Lesion Width (mm)	Axial	6 Months	0.299 ± 0.08	0.291 ± 0.22	0.445 ± 0.29	F(2,7)=0.46	0.649
MRI Lesion Thickness (mm)	Axial	6 Months	0.549 ± 0.15	0.214 ± 0.17	0.545 ± 0.17	F(2,7)=4.94	0.046

Sagittal lesion length showed progressive reduction over time in all groups, with mean improvement rates at six months of 0.488 ± 0.15 in the ESWT group, 0.387 ± 0.23 in the PRP group, and 0.411 ± 0.04 in the PRP+ESWT group, without significant between-group differences (P = 0.814). Similarly, axial lesion width improved across all groups but did not differ significantly among treatments (P = 0.670).

In contrast, axial lesion thickness demonstrated significantly greater improvement among treatment groups at six months (F(2,7) = 4.94, P = 0.046). Greater structural improvement was observed in both the ESWT (0.549 ± 0.15) and PRP+ESWT (0.545 ± 0.17) groups compared with PRP monotherapy (0.214 ± 0.17).

**Figure 2:**
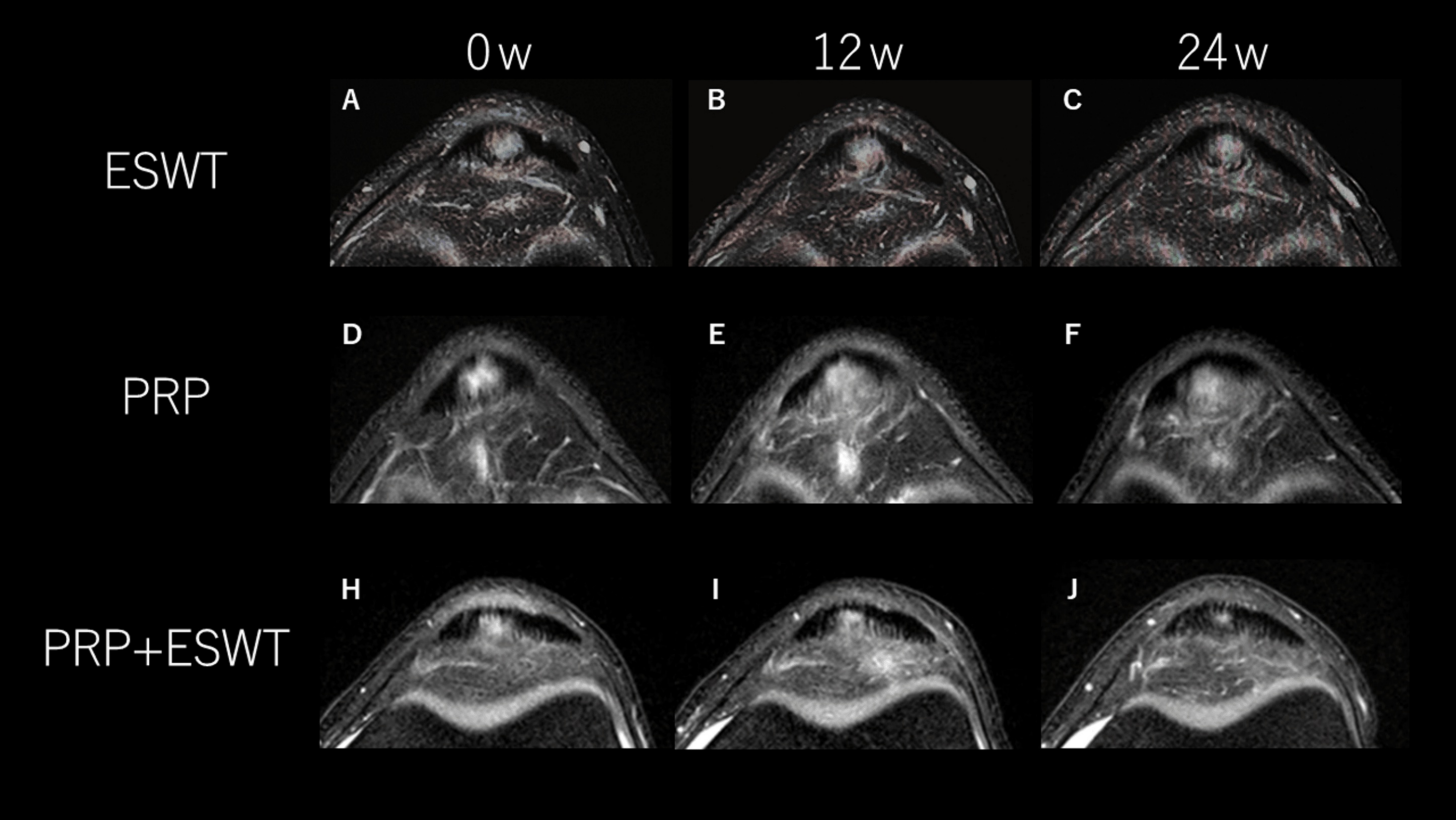
Representative MRI Changes Over Six Months in the ESWT, PRP, and PRP+ESWT Groups Representative axial short tau inversion recovery (STIR) MRI images demonstrating longitudinal changes in intratendinous lesion morphology over a six-month follow-up period. Images were obtained at baseline (0 weeks), 12 weeks, and 24 weeks for each treatment group. (A–C) ESWT group: Minimal morphological change in intratendinous hyperintense signal was observed throughout follow-up. (D–F) PRP group: Partial reduction in intratendinous hyperintensity was observed, although residual signal alteration remained at final follow-up. (H–J) PRP+ESWT group: Apparent reduction in intratendinous hyperintense lesion extent was observed at 12 and 24 weeks. Lesions were defined as intratendinous hyperintense regions on STIR sequences. Representative MRI images are shown for illustrative purposes only. These images are not intended for statistical comparison but to visually demonstrate typical morphological changes observed within each treatment group. Statistical conclusions are based exclusively on quantitative imaging analyses. Abbreviations: ESWT, extracorporeal shock wave therapy; PRP, platelet-rich plasma

Representative STIR MRI findings are presented in Figure 2 to illustrate qualitative morphological changes observed over time. These images are provided for illustrative purposes only, while statistical conclusions are derived from quantitative imaging analyses.

In the ESWT group, intratendinous hyperintense signal demonstrated limited morphological change from baseline to 24 weeks. The PRP group showed partial reduction of signal intensity, although residual abnormalities persisted at final follow-up. In contrast, the PRP+ESWT group tended to demonstrate greater contraction of lesion extent and reduction of intratendinous hyperintensity. These qualitative observations were generally consistent with temporal improvement trends but should be interpreted in conjunction with the quantitative analyses.

Ultrasonographic outcomes

Ultrasonographic parameters revealed significant between-group differences favoring combined therapy (Table 4).

At six months, hypoechoic lesion area demonstrated significantly greater improvement in the PRP+ESWT group (0.874 ± 0.10) compared with ESWT (0.315 ± 0.40) and PRP alone (0.588 ± 0.11) (P = 0.031).

Post-hoc Tukey analysis confirmed significantly greater improvement in the PRP+ESWT group compared with ESWT alone (mean difference +0.56, adjusted P = 0.028) (Table 5).

**Table 5:** Post-Hoc Multiple Comparisons (Tukey Test) (post-hoc performed only when ANOVA significant) Post-hoc pairwise comparisons were conducted using Tukey’s honestly significant difference (HSD) test following significant one-way analysis of variance (ANOVA) results. Mean differences represent between-group differences in percentage improvement from baseline ultrasonographic measurements of lesion size. Ultrasonographic lesion area is expressed in square millimeters (mm²), and lesion length is expressed in millimeters (mm). Adjusted p-values account for multiple comparisons. Abbreviations: ESWT, extracorporeal shock wave therapy; PRP, platelet-rich plasma.

Outcome	Comparison	Mean Difference	Adjusted p-value
US Lesion Area (mm²)	PRP+ESWT vs ESWT	0.56	0.028
	PRP+ESWT vs PRP	0.29	0.071
	PRP vs ESWT	0.27	0.119
US Lesion Length (mm)	PRP+ESWT vs ESWT	0.43	0.012
	PRP+ESWT vs PRP	0.39	0.021
	PRP vs ESWT	0.04	0.842

Similarly, intratendinous lesion length assessed by ultrasonography showed significant intergroup differences (P = 0.015). The PRP+ESWT group demonstrated the greatest improvement (0.777 ± 0.10), significantly exceeding both ESWT alone (mean difference +0.43, adjusted P = 0.012) and PRP alone (mean difference +0.39, adjusted P = 0.021).

No significant difference was observed between PRP and ESWT groups (P = 0.842).

Overall, ultrasonographic findings consistently demonstrated superior morphological recovery in the combined PRP+ESWT group compared with monotherapy.

Return to sport

At six months, no patient reported pain during daily activities, and all returned to sport. Return to pre-injury level was achieved in 50% of the ESWT group, 80% of the PRP group, and 100% of the PRP+ESWT group (P = 0.34). Pain during sport was reported in 50%, 40%, and 67% of the respective groups (P = 0.83). Recurrence occurred in one patient each in the ESWT and PRP groups and in none of the PRP+ESWT group (P = 0.42). No statistically significant intergroup differences were observed (Table 6).

**Table 6:** Six-Month Sports-Related Clinical Outcomes by Treatment Group Categorical sports-related outcomes at six months are presented for extracorporeal shock wave therapy (ESWT), platelet-rich plasma (PRP), and combined PRP plus ESWT groups. Outcomes include absence of pain in daily life, return to sport participation, return to preinjury performance level, presence of pain during sport, and recurrence. Footnote: Values are presented as n(%). † Fisher–Freeman–Halton exact test Values are presented as number (percentage). Between-group comparisons for categorical variables were performed using the Fisher–Freeman–Halton exact test because of the small sample size and expected cell counts less than five. All tests were two-sided, and P < 0.05 was considered statistically significant. The absence of statistically significant differences should be interpreted cautiously due to limited statistical power associated with the small sample size. Abbreviations: ESWT, extracorporeal shock wave therapy; PRP, platelet-rich plasma.

Outcome	ESWT (n=2)	PRP (n=5)	PRP+ESWT (n=3)	P value†
No pain in daily life	2 (100%)	5 (100%)	3 (100%)	—
Return to sport	2 (100%)	5 (100%)	3 (100%)	—
Return to pre-injury level	1 (50%)	4 (80%)	3 (100%)	0.34
Pain during sport	1 (50%)	2 (40%)	2 (67%)	0.83
Recurrence	1 (50%)	1 (20%)	0 (0%)	0.42

Illustrative case

A 21-year-old male decathlete presented with a six-month history of anterior right knee pain aggravated by jumping and acceleration activities and refractory to conservative physiotherapy.

**Figure 3:**
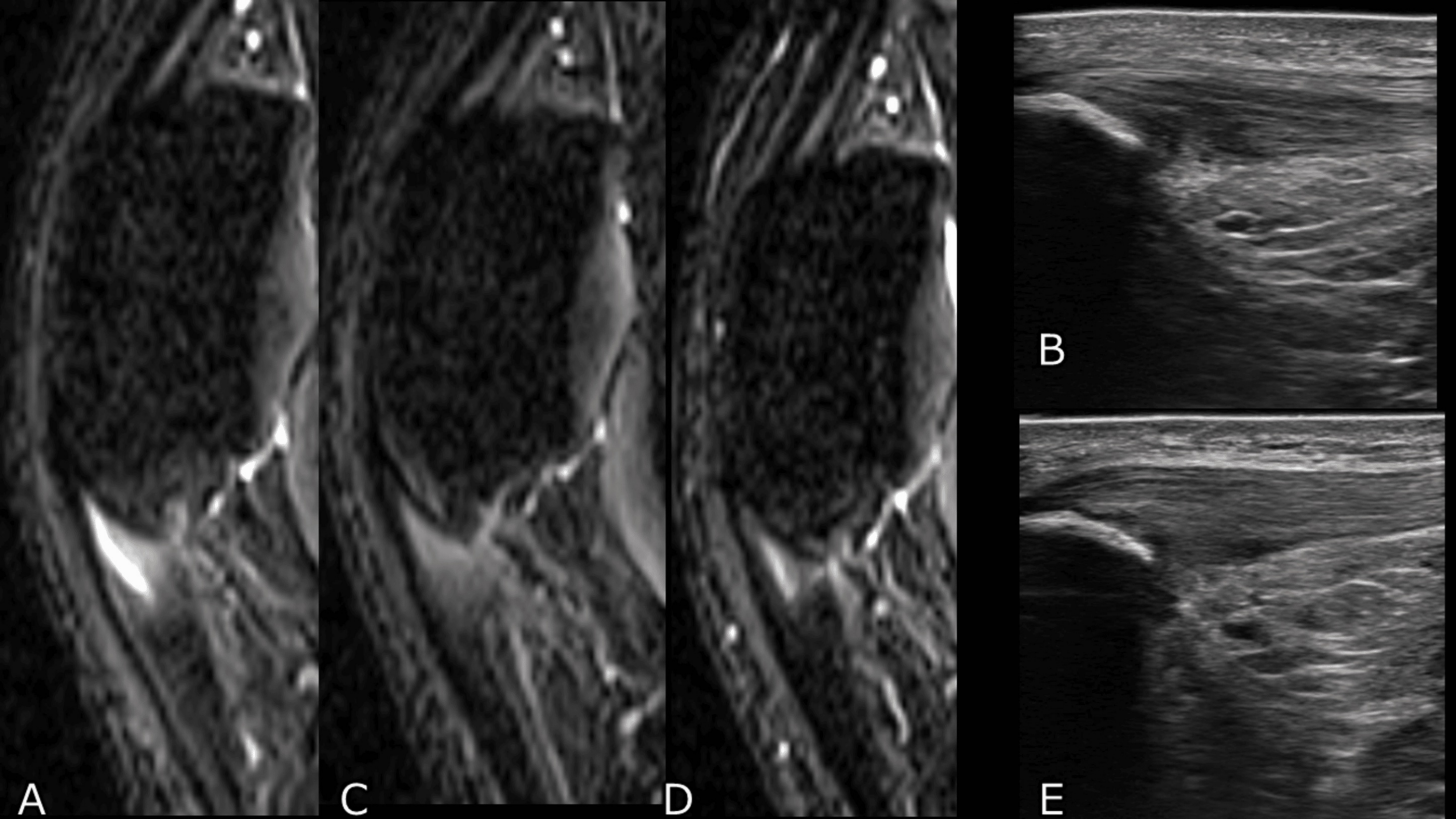
Representative case of a 21-year-old male patient in the PRP plus ESWT group. (A) Baseline short tau inversion recovery (STIR) MRI showing intratendinous hyperintensity at the proximal patellar tendon. (B) Baseline ultrasonography showing tendon thickening and a hypoechoic region. (C) MRI at eight weeks demonstrating reduction of the hyperintense lesion. (D) MRI at six months demonstrating structural improvement. (E) Ultrasonography at 6 months demonstrating remodeling of tendon fiber architecture. Footnote: MRI images illustrate changes in intratendinous signal intensity, whereas ultrasonography demonstrates reduction in hypoechoic regions corresponding to tendon structural improvement. All images are from the same representative patient. Abbreviations: MRI, magnetic resonance imaging, ESWT, extracorporeal shock wave therapy; PRP, platelet-rich plasma.

Physical examination revealed localized tenderness at the proximal patellar tendon. STIR MRI demonstrated a focal intratendinous hyperintense lesion (Figure 3A), and ultrasonography showed tendon thickening with a corresponding hypoechoic area (Figure 3B). The patient was allocated to the combined therapy group according to the study protocol.

Clinical scores improved over the follow-up period. KOOS Total increased from 58 at baseline to 94.8 at three months and was 87.5 at six months. VISA-P improved from 71 to 97, and VAS decreased from 46.5 to 0 at three months and 9.5 at six months.

Graded return to training was initiated at two months, and the athlete resumed competition at three months. Follow-up STIR MRI demonstrated reduction of the intratendinous hyperintense lesion at eight weeks (Figure 3C) and further morphological improvement at six months (Figure 3D). Ultrasonography showed progressive reduction of the hypoechoic area with improved tendon fiber homogeneity (Figure 3E). No recurrence was observed during the six-month follow-up.

## Discussion

In this randomized comparative study, baseline characteristics including age, sex, and body mass index were balanced across groups, supporting internal validity and suggesting that observed between-group differences were plausibly attributable to the interventions.

In analyses of pain and functional outcomes, the PRP plus ESWT group showed numerically greater improvements, although statistically significant intergroup differences were not observed. A plausible explanation is the interaction between biological and mechanical stimuli. ESWT has been reported to suppress pain transmission via modulation of free nerve endings and reductions in neuropeptides such as CGRP and substance P, while also promoting improved perfusion and angiogenesis through upregulation of VEGF and eNOS [18,25]. In addition, ESWT may organize a pro-repair environment via mechanotransduction-mediated intracellular signaling [26]. These mechanisms may enhance responsiveness to the growth factors delivered by PRP.

A potential explanation for the clinical improvements observed in the combined treatment group may be the complementary biological and mechanical mechanisms of PRP and ESWT. PRP provides a high concentration of growth factors, including PDGF, TGF-β, VEGF, and insulin-like growth factor (IGF), which are known to promote tenocyte proliferation, collagen synthesis, and extracellular matrix remodeling. In contrast, ESWT is believed to stimulate mechanotransduction pathways, increase local blood flow, and induce neovascularization, thereby enhancing tendon regeneration and pain modulation. The integration of these biological and mechanical stimuli may therefore create a synergistic therapeutic environment that promotes tendon repair and functional recovery. Consistent with the clinical findings, imaging outcomes demonstrated structural improvement across modalities. Ultrasonographic analyses revealed significant between-group differences in hypoechoic lesion area and intratendinous lesion length, favoring the combined PRP plus ESWT treatment.

Importantly, MRI analysis further demonstrated a significant between-group difference in axial lesion thickness at six months, suggesting that combined therapy may contribute not only to superficial morphological changes detectable by ultrasonography but also to deeper structural remodeling within the tendon matrix.

Growth factors contained in PRP, including PDGF, TGF-β, VEGF, and IGF, are known to promote tenocyte proliferation, collagen synthesis, and extracellular matrix remodeling [27]. ESWT may further potentiate these effects by amplifying growth factor release and transiently increasing cell membrane permeability (sonoporation), thereby improving cellular uptake and downstream signaling [16,17].

The observed improvement in MRI-derived tendon thickness may reflect enhanced extracellular matrix reorganization and normalization of intratendinous structure resulting from synergistic biological and mechanical stimulation.

However, return-to-sport rates at six months did not differ among groups. This may reflect a temporal gap between structural repair and functional readiness, because return to sport depends on multiple factors including neuromuscular control and load tolerance in addition to tissue morphology [28-30]. Furthermore, the uniformly high return-to-sport rate across all groups suggests the possibility of a ceiling effect that may have limited the detection of between-group differences.

Taken together, the present findings indicate that combined PRP and ESWT may facilitate not only symptomatic recovery but also MRI-confirmed structural tendon remodeling in athletes with refractory patellar tendinopathy, supporting a potential synergistic interaction between biological stimulation and mechanotransduction. Rather than acting as purely additive treatments, ESWT may prime the local repair environment and enhance responsiveness to PRP-derived biological signaling, thereby promoting structural improvement and forming the conceptual basis of bio-mechano-therapy.

This study has several limitations. First, the sample size was relatively small, which may have limited the statistical power to detect significant differences between treatment groups. This exploratory pilot study was designed to evaluate feasibility and preliminary treatment effects. Second, clinical and imaging assessments were performed by a single investigator, which may introduce observer bias. Future studies with blinded multi-observer evaluation are warranted. Third, the follow-up period was limited to six months. Longer-term follow-up studies are necessary to evaluate the durability of treatment effects. Finally, although improvements were observed in all treatment groups, some comparisons did not reach statistical significance, which may be related to the limited statistical power associated with the small sample size.

Overall, our findings suggest that bio-mechano-therapy may represent a theoretically coherent and clinically meaningful conservative strategy for refractory patellar tendinopathy and provide a foundation for further development of biologically informed interventions.

## Conclusions

This randomized controlled study investigated the effects of bio-mechano-therapy (combined PRP and ESWT) on the recovery process in athletes with refractory patellar tendinopathy. Baseline characteristics were balanced across groups. The PRP plus ESWT group demonstrated the greatest improvements in pain and functional outcomes and showed the most favorable structural changes on MRI and ultrasonography, including reduction of lesion extent and higher improvement rates. In contrast, return-to-sport rates at six months did not differ among groups.

These findings are consistent with a theoretical framework in which ESWT-mediated neuromodulation and improved perfusion complement PRP-derived growth factor signaling, and the integration of biological and mechanical stimuli may accelerate tendon repair. Early pain suppression and promotion of structural restoration may facilitate the overall recovery process.

In conclusion, bio-mechano-therapy appears to be a promising conservative treatment strategy for refractory patellar tendinopathy and may promote recovery through both symptomatic and structural improvement. Nevertheless, limitations related to sample size, follow-up duration, and lack of direct molecular verification should be acknowledged. Larger multicenter studies incorporating biological assessments are needed to further clarify efficacy and mechanisms.
